# DIESL fuels a DGAT-independent triglyceride synthesis pathway

**DOI:** 10.1093/lifemeta/load039

**Published:** 2023-10-05

**Authors:** Lauren F Uchiyama, Peter Tontonoz

**Affiliations:** Department of Pathology and Laboratory Medicine, Molecular Biology Institute, David Geffen School of Medicine, University of California, Los Angeles, CA 90095, United States; Department of Pathology and Laboratory Medicine, Molecular Biology Institute, David Geffen School of Medicine, University of California, Los Angeles, CA 90095, United States

**Alternative triglyceride (TG) synthesis pathways have yet to be identified in mammalian cells. In a recent article published in *Nature*, Brummelkamp and colleagues reported that the acyltransferase transmembrane protein 68 (TMEM68)/DIESL synthesizes TG in the absence of the canonical enzymes diacylglycerol acyltransferase 1 (DGAT1) and DGAT2.**

**Figure 1:**
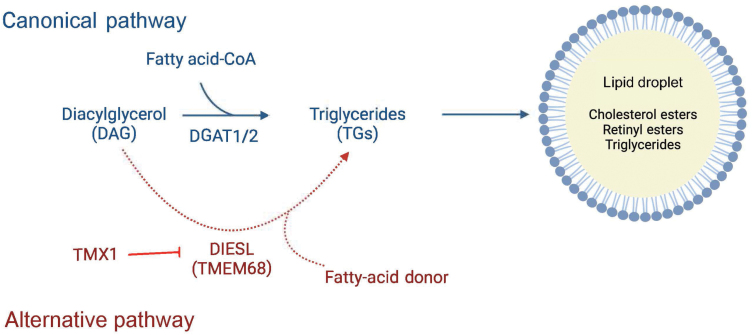
Schematic of acyltransferases involved in triglyceride LD synthesis in mammalian cells. Canonical triglyceride synthesis pathway (blue): DAG is converted into TGs by enzymes DGAT1/2. They use fatty acid-CoA as the acyl donor. Alternative triglyceride synthesis pathway (red): TMX1 negatively regulates DIESL/TMEM68. DIESEL uses an unknown fatty acid donor to produce TGs. Created with Biorender.com.

Triglycerides (TGs) are synthesized in the endoplasmic ­reticulum (ER) and stored in the form of lipid droplets (LDs). The last step of the canonical synthesis pathway esterifies diacylglycerol (DAG) with acyl-coenzyme A (acyl-CoA) to form TGs (Fig. 1). This reaction is catalysed by diacylglycerol acyltransferase 1 (DGAT1) and DGAT2 [1]. Both DGATs have acyltransferase ­activity with varying substrate affinities and specificities, and both ­contribute to LD formation in different physiologic contexts. However, they arise from evolutionarily different families of ­proteins and have low sequence homology. They also have ­different subcellular localization—DGAT1 is restricted to the ER while DGAT2 can localize around LDs [2].

LDs are required to store TGs and prevent the build-up of toxic free-fatty acids (FFAs) in the cytoplasm [2, 3]. The physiologic ­relevance of TG-rich LDs is tissue- and cell-type specific. Following lipolysis in white fat, a proportion of produced FFAs are re-­esterified into TGs. Adipocytes without DGAT1/2 have no detectable LDs, and the cells become more reliant on glucose. *DGAT1* adipose-specific knockout mice are protected from diet-induced obesity, but are also less glucose and insulin tolerant [2]. Adipocytes lacking DGAT also have elevated levels of ER stress in response to stimulated lipolysis, supporting the notion that the esterification of FFAs by DGAT1 serves to protect them from lipotoxicity. In contrast, adipose *DGAT2*-knockout mice show decreased lipogenesis gene expression during fasting, but lack overt metabolic phenotypes [2]. In the liver, hepatocytes transiently store TGs in LDs but also pack lipids into lipoproteins for secretion. Loss of either DGAT1 or DGAT2 alone in hepatocytes reduces mouse liver TG levels. In addition, hepatocyte *DGAT2*-deficient mice show decreased very low-density lipoprotein TG secretion. Early-phase clinical trials inhibiting DGAT2 decreased liver fat content in patients, suggesting that DGAT2 may be effective in preventing metabolic dysfunction-associated steatohepatitis (formally known as non-alcoholic steatohepatitis) [1].

Interestingly, DGATs have been shown to be dispensable for the formation of LDs in certain cell types. Macrophages isolated from *DGAT1/2*-deficient mice are still able to form LDs in the presence of low-density lipoprotein (LDL) cholesterol [4]. Loading these cells with cholesterol also induces TG synthesis, suggesting that DGATs are not required for TG synthesis in this context. Phospholipids can also be used as an acyl-donor to DAG to form TGs [phospholipid diacylglycerol acyltransferase (PDAT) enzymes], but this reaction has only been documented in yeast and plants [5]. Moreover, inhibiting cholesterol and DAG acyltranferases in addition to PDAT in yeast is required to completely prevent LD formation [6, 7]. DAG transacylations can potentially use two molecules of DAGs to yield TGs.

The Brummelkamp group has previously used a haploid cell screening pipeline to identify novel proteins and alternative mechanisms in multiple areas of biology [8]. In their most recent work, McLelland *et al*. [9] sought to identify regulators of LD production using a retroviral gene trap screen in haploid human (HAP1) cells lacking both DGAT1 and DGAT2. LDs were labelled with BODIPY (4-bora-3a,4a-diaza-s-indacene) dye and then monitored with fluorescence-activated cell sorting to quantify TG accumulation in retrovirally transduced cells. Cells were subjected to deep sequencing to identify the causal genes. Thioredoxin-related transmembrane protein 1 (TMX1) emerged as a dominant hit in the screen. To validate this hit, the authors performed both microscopy and thin-layer chromatography (TLC) on *DGAT1/2*-knockout cells in the presence or absence of TMX1. Remarkably, they found increased LD number and TG levels in *TMX1/DGAT*-deficient cells, suggesting that TMX1 was actively suppressing TG accumulation in cells that cannot synthesize TGs by the canonical enzyme pathway (*DGAT1/2* knockout).

To determine the identities of protein(s) that TMX1 negatively regulates, the authors performed two additional screens. In the first, they loaded wild-type HAP1 cells (which are primarily dependent on DGAT1/2 for TG synthesis) with oleic acid. In the second, they loaded HAP1 cells lacking the major negative regulator of TG synthesis that they had identified (*TMX1*-knockout). In wild-type HAP1 cells loaded with oleic acid, *DGAT1* was found to be the strongest driver of LD accumulation, while the well-known inducer of adipose triglyceride lipase (ATGL), ABHD5 (α/β hydrolase domain-containing protein 5, also known as CGI-58), was identified as an inhibitor. These expected hits confirmed the robustness of the authors’ approach. When the results from the wild-type and *TMX1*-knockout HAP1 screens were compared, transmembrane protein 68 (TMEM68) emerged as the number one hit. In recognition of its newly revealed function described below, the authors renamed TMEM68 as DGAT1/2-independent enzyme synthesizing storage lipids (DIESL).

To further study the ability of TMX1 to negatively regulate TG accumulation through DIESL, the authors performed TLC on extracts of *DGAT1/2*-knockout cells in the presence or absence of TMX1, and the presence or absence of DIESL. Cells lacking DIESL, TMX1, and DGATs had no LDs. In the absence of oleic acid, *TMX1*-knockout cells accumulated LDs (as measured by microscopy), but *TMX1/DIESL*-double knockout cells did not. These data suggest that DIESL is required for the effect of TMX1 on LD accumulation. The authors further assessed the effect of *DIESL* knockout in three different cell lines under conditions of DGAT inhibition and found that TG levels were reduced by 15%−30%. Thus, the DIESL pathway accounts for a small but measurable proportion of TG synthesis in several mammalian cell lines.

Previous work has suggested that both TMX1 and DIESL are localized to the ER. DIESL is an integral ER membrane protein, similar to DGAT1, and remains in the ER in the presence of LDs [10]. Importantly, the ER is the subcellular localization of TG synthesis and LD production [1]. The authors explored the interaction of TMX1 and DIESL with biochemical assays. They used 1% paraformaldehyde to crosslink endogenous proteins and identified a TMX1-DIESL heterodimer by immunoblotting. TMX1 and DIESL were further shown to co-immunoprecipitate, but only in a 1% Tween20 detergent-containing lysis buffer, suggesting that they may interact in the membrane. Further studies need to be conducted to determine the exact nature of their interaction.

McLelland *et al*. [9] next used sequence homology analysis and AlphaFold structural prediction software to infer the presence of an acyltransferase-like domain in DIESL, which is consistent with previous findings. Chang *et al*. [10] also performed phylogenetic and sequence analysis of murine TMEM68 showing that this family is evolutionarily most similar to the DGAT2 family of proteins, and may have both glycerophospholipid and DGAT domains. Based on this model, McLelland *et al*. [9] created a cata­lytically dead mutant H130A and showed that it was unable to promote the generation of LDs in HAP1 cells. Shotgun lipidomics were performed on *DGAT1/2*, *TMX1*, and *DIESL* quadruple knockout cells reconstituted with either wild-type DIESL or the H130A mutant to determine DIESL’s effects on the cellular lipidome, independent of DGATs. Wild-type DIESL expression increased TGs and decreased the DAG and phosphatidylcholine percentages of the lipidome.

Having demonstrated the effects of DIESL on static TG levels by TLC, lipidomics, and microscopy, the authors proceeded to measure TG synthesis directly. Using both radiolabeled DAG in lysate assays and nitrobenzoxadiazole-labelled fluorescent-DAG in intact cells to measure DAG acylation, they showed that wild-type DIESL increased the rate of DAG acylation into TG, while the H130A mutant did not. These data suggest that DIESL is an acyltransferase that promotes TG synthesis independent of DGAT enzymes in the absence of its negative regulator TMX1. It is unclear what the acyl donor of this enzyme is (acyl-CoA, DAG, or phospholipid) without tracing potential donors, but it will be exciting to find out the source.

A critical outstanding question at this point in the story is how the DIESL pathway for TG synthesis affects systemic metabo­lism and physiology. Global *DIESL*-knockout mice exhibited decreased plasma TGs, and a lower body weight compared to the controls. These effects were most prominent immediately after the age of weaning, before which time the animals are consuming large amounts of lipids from milk. These phenotypes disappeared as the mice approached adulthood in males, but not in females. Whether these observations are related to a change in dietary composition from fat to carbohydrates after weaning or to a requirement for extra TGs during the acute growth stage is a question for future investigation. Assessment of body composition and respiratory exchange ratio in a pilot cohort of mice suggested that *DIESL*-knockout mice were smaller and more reliant on glucose as an energy source. This finding supports a physiologi­cal role for DIESL in mammalian TG metabolism.

Several interesting but tangential observations related to LD biology emerged from this work. First, the LD-associated genes *BSCL2* (seipin, Bernardinelli-Seip congenital lipodystrophy 2) and *HILPDA* (hypoxia-inducible lipid droplet associated) were more highly upregulated in oleic acid-loaded wild-type HAP1 cells compared to those lacking TMX1. This observation suggests that the alternative TG synthesis pathway is less ­seipin-dependent, at least at the gene expression level. Second, DGAT1 but not DGAT2 seemed to be a positive regulator of LD synthesis in oleic acid-loaded wild-type HAP1 cells. This finding is in line with data from multiple groups showing DGAT1 is more crucial for LD synthesis during fasting and has a higher substrate affinity than DGAT2. Third, the sterol regulatory element-binding protein 2 (SREBP2) pathway for cholesterol synthesis and acyl-CoA: cholesterol acyltransferase 1 (ACAT1, the enzyme for cholesterol esterification) were induced in both wild-type and *TMX1*-deficient cells. This observation may suggest that cholesterol-ester-containing LDs are able to compensate for the absence of TG-rich LDs, or that cholesterol esters (CEs) are required for the formation of any LD-containing triglycerides. This finding is also reminiscent of the prior observation that *DGAT1/2* knockout macrophages loaded with LDL can synthesize some triglycerides, potentially from ACAT1/2 activity [4]. Fourth, certain fatty acid synthesis enzymes, including Gpat3 (glycerol-3-phosphate acyltransferase 3), Scd (stearoyl-CoA-desaturase), and Fasn (fatty acid synthase), were selectively induced in *TMX1*-deficient, but not wild-type cells. What transduces this signal?

Collectively, the study of McLelland *et al*. [9] provides compelling evidence for the existence of a previously unappreciated alternative mechanism for TG production in mammals. Like most innovative work, their findings generate many questions. For example, how does this alternative TG production process affect LD synthesis and morphology at the molecular level? What molecule is the acyl donor for DIESL and can the reaction be recontsituted *in vitro* with isolaed components? Most importantly, in what tissues and in which physiological or pathological contexts is this pathway activated? The HAP1 cells are a single clone of the almost-haploid KBM7 (chronic myelogenous leukemia) cell line infected with a retrovirus cocktail containing SOX2 (SRY-box transcription factor 2), c-MYC (Avian myelocytomatosis virus oncogene cellular homolog), OCT4 (Octamer-binding transcription factor 4), and KLF4 (Kruppel-like factor 4) to induce pluripotency. Thus, the present screen was performed in a hyper-proliferative/stem cell-like condition that may require more TGs. Perhaps the alternative pathway is activated in cancer, tissues with short half-lives, and conditions of extreme nutrient deprivation or DGAT suppression. Chang *et al*. [10] suggest that TMEM68/DIESL is most highly expressed in the mouse brain, suggesting particular rele­vance in this context. Nonetheless, this article has uncovered new and provocative biology relevant to lipid metabolism and metabolic disease, and has opened the door to exciting avenues of future investigation.
